# Efficacy and Safety of Multihole Partially Covered Self‐Expandable Metal Stents for Distal Malignant Biliary Obstruction: A Single‐Center Retrospective Study

**DOI:** 10.1002/deo2.70168

**Published:** 2025-06-22

**Authors:** Kengo Matsumoto, Kazuhide Iwasa, Asuka Watanabe, Hiroki Takiyama, Satoru Okabe, Naoto Osugi, Dai Nakamatsu, Masashi Yamamoto, Shiro Hayashi, Koji Fukui, Tsutomu Nishida

**Affiliations:** ^1^ Department of Gastroenterology Toyonaka Municipal Hospital Osaka Japan; ^2^ Department of Gastroenterology and Internal Medicine Hayashi Clinic Osaka Japan

**Keywords:** cholecystitis, distal malignant biliary obstruction, ERCP complications, pancreatitis, self‐expandable metal stents

## Abstract

**Background:**

Covered self‐expandable metal stents (SEMS) are the standard for managing unresectable distal malignant biliary obstruction (DMBO), as they prolong the time to recurrent biliary obstruction (TRBO). However, fully covered SEMS (FCSEMS) increases the risk of cholecystitis and pancreatitis. This exploratory study evaluated a novel multihole partially covered SEMS (MHSEMS) designed to reduce these risks.

**Methods:**

The clinical data of 26 DMBO patients treated with MHSEMSs were retrospectively compared with those of 63 patients treated with FCSEMSs between April 2018 and October 2024. The outcomes included clinical success, early complications, and recurrent biliary obstruction (RBO).

**Results:**

The baseline characteristics, including age (median 78 years), sex distribution (55.2% vs. 57.7% male), BMI (20.5 vs. 19.8), tumor size (27 mm vs. 30 mm), and stricture length (20 mm vs. 19.5 mm), were comparable between the groups. Procedural factors, including initial papillary cannulation (34.9% vs. 26.9%) and pancreatography (12.9% vs. 15.4%), were also similar in terms of incidence. Early complications were less common in the MHSEMS group (7.7% vs. 23.8%), with no cases of cholecystitis observed. Fewer early complications were observed with MHSEMS, suggesting potential clinical benefits. The RBO rates (7.7% vs. 15.9%, *p* = 0.28) and median TRBO (151 vs. 141.5 days, *p* = 0.87) were also comparable.

**Conclusion:**

Although the differences in outcomes were not statistically significant, the incidence of early complications was lower, especially for cholecystitis, with the MHSEMS in the management of DMBO. Larger prospective studies are needed to confirm these preliminary findings.

## 1 Introduction

Distal malignant biliary obstruction (DMBO), often linked to advanced pancreatic or biliary cancers, poses a major clinical concern. [1]. This condition leads to jaundice, pruritus, and increased cholangitis risk, necessitating prompt biliary drainage. Endoscopic retrograde cholangiopancreatography (ERCP) with self‐expanding metallic stent (SEMS) placement is the standard palliative treatment for DMBO [2]. Fully covered self‐expanding metallic stents (FCSEMSs) are advantageous because they prevent tumor ingrowth and are removable. Compared with uncovered or partially covered SEMSs, FCSEMSs prolong the time to recurrent biliary obstruction (TRBO) [3]. This advantage improves quality of life and reduces repeat procedures.

Despite these benefits, FCSEMSs have notable drawbacks. Their full coverage may block the cystic duct or pancreatic duct, increasing early complication risks, including cholecystitis and post‐ERCP pancreatitis (PEP). These complications worsen patient outcomes and burden healthcare systems, making them a significant concern [4, 5].

To address these challenges, a novel multihole SEMS (MHSEMS) was developed. It features small lateral holes designed to maintain bile flow and reduce the risk of cystic and pancreatic duct obstruction, thereby potentially lowering the incidence of cholecystitis and pancreatitis while preserving the antitumor benefits of FCSEMS. This exploratory study aims to assess the safety and efficacy of MHSEMS, focusing on whether it can reduce early complications without compromising performance.

## 2 Patients and Methods

### 2.1 Study Design

This was a single‐center retrospective study conducted between April 2018 and October 2024. Patients with DMBO and symptoms such as jaundice or elevated bilirubin and transaminases were included.

#### 2.1.1 Characteristics of the SEMS

**FIGURE 1:**
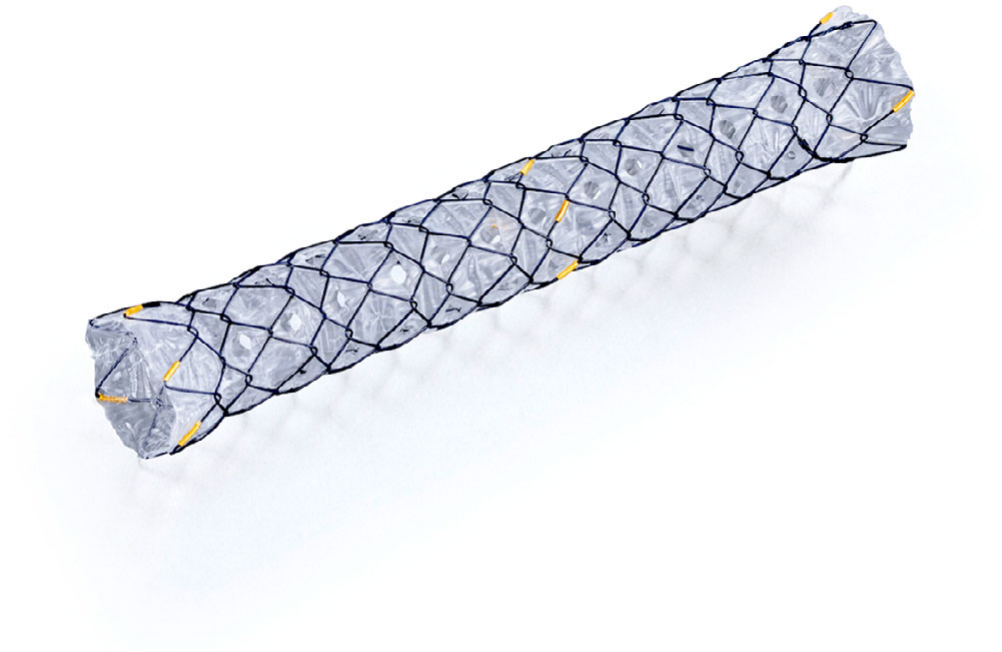
The multihole self‐expandable metal stent.

The FCSEMS used was either a HANAROSTENT Biliary Full Cover NEO (Boston Scientific Japan, Tokyo) or a BONASTENT Biliary (Medico's Hirata Inc., Japan, Tokyo). The MHSEMS used was a HANAROSTENT Biliary Multihole NEO (Boston Scientific Japan, Tokyo) (Figure 1). Like an FCSEMS, this stent is composed of nitinol wire and is fully covered in a silicone membrane. However, it is termed ‘partially covered’ due to multiple 1.8 mm lateral holes, allowing flow between the stent lumen and bile duct wall. The 6, 7, and 8 cm stents had 66, 78, and 90 holes, respectively, in six equally spaced rows.

### 2.2 Patients

This retrospective study included all consecutive patients who underwent SEMS placement for unresectable DMBO at Toyonaka Municipal Hospital between April 2018 and October 2024. Patients with various disease stages, including those with locally advanced or metastatic disease, as well as those receiving chemotherapy or best supportive care, were included. No patients in this cohort had received radiotherapy, although this was not an exclusion criterion. There were no specific exclusion criteria; all patients who underwent SEMS placement for DMBO during the study period were analyzed.

### 2.3 ERCP Procedure and Stent Placement

Biliary cannulation was performed at the physician's discretion using any type of ERCP catheter or guidewire. After DMBO was confirmed via cholangiography, a guidewire was placed, followed by endoscopic papillotomy. Cases categorized as “cholecystogram yes” were those in which the gallbladder was incidentally opacified during cholangiography performed to assess the extent of common bile duct stenosis.

When the gallbladder was opacified, the contrast medium was aspirated as much as possible before stent placement. Nasobiliary drainage tubes or plastic stents (PS) were placed in 60 patients (67%) prior to SEMS placement, per guidelines [1]. These were used for drainage before definitive diagnosis and treatment planning. Patients underwent placement of an FCSEMS from April 2018 to September 2023 and an MHSEMS from October 2023 to October 2024.

The procedures were performed at a Japan Gastroenterological Endoscopy Society (JGES)‐certified teaching hospital (No. 1239), where trainees were supervised by experts to ensure high‐quality surgery and few complications. Duodenoscopes (TJF260V or TJF‐Q290V: Olympus Optical Co., Tokyo, Japan; ED‐580T: Fujifilm Co., Tokyo, Japan) were used for all procedures.

### 2.4 Outcomes

The primary outcome was the incidence of early adverse events, defined as complications unrelated to recurrent biliary obstruction (RBO), such as post‐ERCP pancreatitis, cholangitis, or perforation. The secondary outcomes included the incidence of RBO, which was defined as stent occlusion or symptomatic migration on the basis of the 2024 Tokyo criteria [6]. The time to RBO (TRBO) was calculated from SEMS placement to RBO onset. The median TRBO is reported only for cases of RBO. This was due to early censoring from transitions to best supportive care (BSC), limiting accurate TRBO estimation. As a result, the TRBO may exceed the overall median observation period. Technical success was defined as proper stent placement at the intended location. Clinical success was defined as cholangitis resolution or effective biliary drainage, indicated by decreased bilirubin and liver enzymes within 7 days. The duodenal invasion was defined as endoscopic evidence of tumor infiltration into the duodenum. The severity of post‐ERCP pancreatitis (PEP) was assessed according to the consensus classification by Cotton et al. [7].

### 2.5 Ethical Consideration

The study complied with the Declaration of Helsinki and was approved by the Ethics Committee of Toyonaka Municipal Hospital (2024‐12‐4). The requirement for informed consent was waived due to its retrospective nature.

### 2.6 Statistical Analysis

Continuous variables are presented as medians with interquartile ranges (IQRs) and categorical variables as frequencies and percentages. Categorical variables were analyzed using chi‐square or Fisher's exact test, depending on expected cell counts, and the Mann–Whitney U test for continuous variables. As an exploratory study with a limited sample size, no formal sample size calculation was performed. Univariate logistic regression analysis was conducted to identify potential risk factors for early adverse events. Multivariate analysis was not performed due to a few events, which could compromise model stability. *p*‐Value < 0.05 indicated statistical significance. Statistical analyses were performed via JMP statistical software (version 17.1.0; SAS Institute, Inc., Cary, NC, USA).

## 3 Results

### 3.1 Study Population

A total of 63 patients underwent FCSEMS placement for DMBO between April 2018 and September 2023, whereas 26 patients underwent MHSEMS placement between October 2023 and March 2024.

### 3.2 Baseline Characteristics

The baseline characteristics were compared between the two groups (Table 1). The median age of the patients was 78 years in the FCSEMS group and 78.5 years in the MHSEMS group (*p* = 0.69). The proportion of males was 55.2% in the FCSEMS group and 57.7% in the MHSEMS group (*p* = 0.85). Primary disease: pancreatic cancer in 52 and 22 patients, and nonpancreatic cancer in 11 and four patients in FCSEMS and MHSEMS groups, respectively (*p* = 0.1211). Pancreatic cancer stages were distributed as follows: 1/7/3/8/33 patients in the FCSEMS group, and 0/8/3/6/5 patients in the MHSEMS group. Stage IV disease was more common in the FCSEMS group. The median BMI: 20.5 versus 19.8 (*p* = 0.94); tumor size: 27 versus 30 mm (*p* = 0.80); stenosis length: 20 versus 19.5 mm (*p* = 0.80). The duodenal invasion was seen in 19.1% of FCSEMS cases and 15.4% of MHSEMS cases (*p* = 0.68). Cystic duct involvement was 22.2% in the FCSEMS group and 26.9% in the MHSEMS group (*p* = 0.63). In all patients, the orifice of the cystic duct was covered by the SEMS. Diclofenac use during ERCP was comparable between groups.

**TABLE 1:** Baseline characteristics.

	FCSEMS	MHSEMS	*p*‐Value
Number of patients, n	63	26	
Age years, median (IQR)	78 (73–84)	78.5 (75.3–83.3)	0.70
Sex, male, *n* (%)	35 (55.2)	15 (57.7)	0.85
BMI	20.5 (18.3–22.2)	19.8 (18.4–21.4)	0.95
History of cholecystectomy, Yes, *n* (%)	0	0	
Gallstones, Yes, *n* (%)	6(9.52%)	4(15.38%)	0.44
Primary disease (Pancreatic cancer/Biliary cancer/other cancer)	52/9/2	22/1/3	0.12
Pancreatic cancer Stage (I / IIa / IIb / III / IV)	1/7/3/8/33	0/8/3/6/5	
Main pancreatic duct obstruction, Yes, *n* (%)	41 (65.1%)	18 (69.2%)	0.71
Tumor involvement of orifice of the cystic duct, Yes, *n* (%)	14 (22.2%)	7 (26.9%)	0.63
Tumor size (mm), median (IQR)	27 (20–33)	30 (24.8–32)	0.80
Stenosis length (mm), median (IQR)	20 (14–25)	19.5 (15.8–25)	0.69
Duodenal invasion, Yes, *n* (%)	12 (19.05%)	4 (15.38%)	0.68
Previous post‐ERCP pancreatitis, Yes, *n* (%)	0 (0%)	0 (0%)	
Cholangitis within the last week, Yes, *n* (%)	14 (22.2%)	5 (19.2%)	0.75
Previous gallbladder drainage, Yes, *n* (%)	1 (1.59%)	1 (3.85%)	0.53

MHPCSEMS: multihole partially covered self‐expandable metallic stent, FCSEMS; fully covered self‐expandable metallic stent, IQR: interquartile range, BMI: body mass index, ERCP: endoscopic retrograde cholangiopancreatography.

### 3.3 ERCP Findings and Procedure Details

ERCP findings and procedures were comparable (Table 2). The proportion of patients with naïve papilla was slightly greater in the FCSEMS group (34.9% vs. 26.9%, *p* = 0.46). Endoscopic sphincterotomy (EST) was performed in all patients during the initial ERCP session. The median cannulation time was 2 minutes in both groups (*p* = 0.31). Pancreatography was performed in 12.9% (FCSEMS) and 15.4% (MHSEMS) (*p* = 0.76). Cholecystography was performed in 25.8% (FCSEMS) and 23.1% (MHSEMS) (*p* = 0.78). Stents measuring 60 mm in length were predominantly used in both groups.

**TABLE 2:** Endoscopic retrograde cholangiopancreatography (ERCP) findings and endoscopic procedure.

	FCSEMS	MHPCSEMS	*p*‐Value
Naïve papilla, *n* (%)	22 (34.9)	7 (26.9)	0.46
Previous biliary plastic stent placement, Yes, n (%)	41 (65.1%)	19 (73.1)	0.46
Canulation time, median (IQR)	2 (1–7)	2 (1–4.25)	
Canulation method (DC/wire‐guided/PGW/precut), *n*	32/21/2/2	17/9/0/0	0.32
Premedication, diclofenac			0.41
None/25 mg/50 mg, *n* (%)	6/32/25	2/11/13	
Procedure time, min	25 (15.8–32.3)	21 (12.8–29.3)	0.76
Pancreatography (%), Yes, *n* (%)	8 (12.9)	4 (15.4)	0.19
Guidewire inserted into the pancreatic duct, Yes, *n* (%)	8 (12.9)	3 (11.5)	0.76
Cholecystogram, Yes, *n* (%)	16 (25.8)	6 (23.1)	0.86
SEMS length 60/70/80 mm, *n*	40/13/9	20/5/1	0.79
			0.32

ERCP: endoscopic retrograde cholangiopancreatography, MHPCSEMS: multihole partially covered self‐expandable metallic stent, FCSEMS: fully covered self‐expandable metallic stent, IQR: interquartile range, DC: direct cannulation, PGW: pancreatic guide wire, SEMS: self‐expandable metallic stent.

### 3.4 Outcomes of SEMS Placement and the Incidence of Early Adverse Events

The technical success rate was 100% in both groups, and the clinical success rates were similarly high (96.2% for the MHSEMS group vs. 92% for the FCSEMS group, *p* = 0.67). The incidence of early adverse events, while not significantly different, was markedly lower in the MHSEMS group (7.7% vs. 23.8%). Pancreatitis occurred in 3.9% of MHSEMS cases, presenting as one mild case, and in 11.1% of FCSEMS cases, including six mild and one severe case. No severe pancreatitis occurred in the MHSEMS group. Acute cholecystitis occurred in 11.1% of FCSEMS cases but was absent in the MHSEMS group. Among seven FCSEMS patients with cholecystitis, four were managed conservatively, one required percutaneous transhepatic gallbladder drainage (PTGBD), and two needed stent removal.

The median observation period was similar (103 days for the MHSEMS vs. 95 days for the FCSEMS [*p* = 0.85]). RBO rates were lower with MHSEMS (7.7% vs. 15.9%, *p* = 0.28), though not significant (Table 3). The median TRBO was comparable (141.5 days for FCSEMS vs. 151 days for MHSEMS, *p* = 0.87). The most common causes of RBO differed: tumor overgrowth in the FCSEMS group (three patients) and food impaction in the MHSEMS group (one patient) (Table 3). One case of stent migration was observed in the MHSEMS group.

**TABLE 3:** Outcomes of self‐expandable metallic stent (SEMS) placement.

	FCSEMS (*N* = 63)	MHPCSEMS (*N* = 26)	*p*‐Value
Technical success, *n* (%)	63 (100)	26 (100)	
Clinical success, *n* (%)	58 (92)	25 (96.2)	0.46
Early adverse events, Yes, *n* (%)	15 (23.8)	2 (7.7)	0.14
Pancreatitis, *n* (%)	7 (11.1)	1 (3.9)	0.67
Mild/ severe, *n* (%)	6/1	1/0	
Cholecystitis, *n* (%)	7 (11.1)	0 (0)	0.10
Conservative/ PTGBD/ Stent removal, *n* (%)	4/1/2	N.A.	
Nonocclusive cholangitis, *n* (%)	1 (1.6)	0 (0)	1.0
Fever, *n* (%)	0 (0)	1 (3.9)	0.29
Observation period, median (IQR)	95 (30.5–190.5)	103 (36–211)	0.58
RBO, *n* (%)	10 (15.9)	2 (7.7)	0.50
TRBO median (IQR)*	141.5 (78.9–208)	151 (138–164)	0.88
Food impaction, *n*	2	1	
Migration, *n*	0	1	
Tumor overgrowth, *n*	3	0	
Nonocclusive cholangitis, *n*	1	0	
Hemorrhage, *n*	2	0	
Pancreatic Pseudocyst, *n*	1	0	
Biliary debris, *n*	1	0	

SEMS: self‐expandable metallic stent; MHPCSEMS: multihole partially covered self‐expandable metallic stent; FCSEMS; full‐covered self‐expandable metallic stent; PTGBD: percutaneous transhepatic gallbladder drainage, N.A: not applicable, IQR: interquartile range; RBO: recurrent biliary obstruction; TRBO: time to recurrent biliary obstruction.

*The median time to recurrent biliary obstruction (TRBO) represents the median for patients with RBO, not the entire cohort.

### 3.5 Univariate Analyses for Early Adverse Events

Univariate analyses did not identify significant risk factors for early adverse events. The use of MHSEMS and omission of cholecystogram were borderline protective (Table 4). Early adverse events did not differ significantly between pancreatic and nonpancreatic cancer patients.

**TABLE 4:** Univariate analyses for early adverse events.

		Univariate		
	**Reference**	**Odds ratio**	**95%**	***p*‐Value**
Sex, male	Female	1.14	0.39–3.34	0.81
BMI, under 22	Over 22	1.67	0.43–6.47	0.46
Pancreatic cancer, no	Yes	2.58	0.75–8.92	0.13
Main pancreatic duct obstruction, no	Yes	2.02	0.69–5.93	0.20
Premedication, diclofenac, no	Yes	1.71	0.40–7.29	0.47
Pancreatography, yes	No	1.48	0.35–6.17	0.59
Cannulation time, over 5 minute	Less than 5 min	1.88	0.60–5.86	0.28
Naïve papilla, yes	No	1.16	0.38–3.53	0.79
Cholecystogram, yes	No	2.61	0.85–8.02	0.099
Type of SEMS, FCSEMS	MHPCSEMS	3.75	0.79–17.75	0.096

HR: Hazard ratio; BMI: body mass index; FCSEMS: fully covered self‐expandable metallic stent; MHPCSEMS: multihole partially covered self‐expandable metallic stent.

## 4 Discussion

In this study, we evaluated the clinical outcomes of MHSEMS in patients with DMBO, focusing on its early complication rate relative to FCSEMS. Our findings suggest that MHSEMS may be safer than FCSEMS, with fewer early complications and similar efficacy in managing RBO. Comparable baseline characteristics enhance the reliability of differences, minimizing confounding by patient factors. Our institution's post‐ERCP pancreatitis rate of 3.9% (95% confidence interval: 3.02%–5.07%) [8] serves as a quality indicator.

A recent study conducted in a high‐volume cancer center and our hospital, a regional community hospital, revealed that the use of MHSEMSs to manage DMBO provided favorable outcomes [9]. Clinical and technical success rates were also comparable. Notably, MHSEMSs were less likely to migrate than FCSEMSs were. Despite the greater median age of our patient cohort (78 vs. 67 years), which better reflects real‐world clinical practice, the outcomes remained consistent. Our study also included patients with biliary tract cancer, expanding the applicability of these findings beyond pancreatic cancer alone. These results suggest that MHSEMS provides robust and reproducible performance in both specialized hospitals and general hospitals. Though prospective trials are needed, the consistent outcomes support the generalizability of MHSEMS.

Comparisons between the UCSEMS and FCSEMS revealed that, because of its fully covered design, an FCSEMS prevents tumor ingrowth and is therefore known for prolonging TRBO, exhibiting greater overall utility [3]. Consistent with prior studies comparing UCSEMSs and FCSEMSs [3], our findings suggest that modifications in stent design, such as the perforated covering of MHSEMS, may mitigate complications while preserving efficacy. With respect to cholecystitis, the UCSEMS is advantageous because it does not obstruct the cystic duct [4, 5]. Consistent with prior reports, the MHSEMS did not lead to any cases of cholecystitis, highlighting its potential to mitigate cystic duct obstruction, a known limitation of the FCSEMS.

A few reports have revealed that the risk factors for cholecystitis are tumor involvement in the orifice of the cystic duct, contrast agent leakage into the gallbladder, and gallstones [5, 10]. The risk factors for the formation of gallstones include potential infection of the bile and impaired contraction of the gallbladder [11, 12] These factors may contribute to the development of cholecystitis after SEMS placement. In our cohort, there were 6 cases of gallstones in the FCSEMS group and 4 cases in the MHSEMS group. Among the seven patients in the FCSEMS group with cholecystitis, one had gallstones. Interestingly, most cases of cholecystitis were observed in patients without gallstones, suggesting that factors other than gallstone presence, such as cystic duct obstruction or contrast leakage, may play a more prominent role. In our cohort, the presence or absence of gallstones did not appear to significantly influence the frequency of cholecystitis after SEMS implantation. According to the univariate analysis, both the use of MHSEMS and the omission of cholecystography prior to stent placement were associated with a trend toward a lower incidence of cholecystitis; however, these differences did not reach statistical significance (*p* = 0.096 and *p* = 0.099, respectively). Although these factors demonstrated a trend toward reduced cholecystitis risk, the lack of statistical significance warrants caution. Further large‐scale studies are warranted to validate these associations. While the lateral holes in MHSEMSs may contribute to a reduction in cholecystitis risk by preserving cystic duct patency, precise control of their alignment with the cystic duct during stent deployment remains challenging. In this study, no specific adjustments were made to optimize the positioning of the side holes.

Given the small sample size and exploratory nature, larger studies are needed to clarify the relationships among SEMS design, gallstone presence, and the risk of cholecystitis.

In cases of pancreatitis, an FCSEMS is likely to compress the pancreatic duct orifice due to its covered design. Previous studies revealed no significant difference in the incidence of pancreatitis between UCSEMSs and FCSEMSs [13]. In this study, pancreatitis was less common with the MHSEMS (3.9%) than with the FCSEMS (11.1%). All cases in the MHSEMS group were mild, whereas one severe and six mild cases occurred in the FCSEMS group. Tumor invasion into the main pancreatic duct may influence the development of pancreatitis, and dilatation of the main pancreatic duct was observed in 65.1% of the FCSEMS group and 69.2% of the MHSEMS group, with no statistically significant difference between the groups (*p* = 0.71). Although a previous study reported no significant difference in the incidence of post‐ERCP pancreatitis between the two stents, their findings showed a similar trend, with pancreatitis occurring in 3.9% of patients in the MHSEMS group and 13.2% in the FCSEMS group [9]. These results, together with our findings, suggest that with a larger sample size, the difference may become statistically significant. These findings suggest that the MHSEMS may not only reduce the incidence of pancreatitis but also potentially help prevent severe cases. The FCSEMS may compress the pancreatic duct orifice, whereas MHSEMS's perforated design likely reduces pressure and prevents blockage, lowering pancreatitis risk. Overall, the incidence of early complications was notably lower with the MHSEMS (7.7% vs. 23.8%).

SEMSs are increasingly used for preoperative drainage in patients with resectable cancers [2]. Reducing the incidence of stent‐related complications is crucial for optimizing chemotherapy and surgical outcomes. The reduced incidence of complications with the MHSEMS suggests that it may be advantageous in these settings.

The mechanism by which the MHSEMS reduces RBO rates likely involves its covered design, whereas its multiple small holes allow limited growth. This minor ingrowth may anchor the stent, reducing the risk of migration [9]. In contrast, FCSEMSs are associated with significantly higher migration rates than UCSEMSs, as stent migration remains a major cause of RBO with FCSEMSs [14].

In this study, the relatively short follow‐up period precluded a comprehensive evaluation of TRBO for all patients. Among those with RBO, no significant difference in TRBO was observed between the two groups. This stent is unlikely to migrate because its design allows the ingrowth of tissue through multiple small side holes along the covering membrane. However, one case of RBO due to stent migration occurred with the MHSEMS, indicating that while its perforated design reduces migration risk, it may not completely prevent migration to the same extent as the UCSEMS does. To prevent stent migration further, the size and number of side holes could be increased.

Although UCSEMSs can be removed in cases of RBO, their extraction is generally more challenging than that of FCSEMSs [14, 15]. The presence of ingrown tissue in the small side holes of MHSEMS may complicate its removal. However, in our study, one patient required early MHSEMS removal due to complications, and removal was performed successfully without difficulty.

This study has several limitations. First, the sample size, particularly in the MHSEMS group, limits the statistical power. While this study was intended as a preliminary investigation of the utility of the MHSEMS, larger multicenter studies are needed to confirm these findings. Second, the follow‐up period was relatively short, restricting the ability to evaluate long‐term outcomes such as late RBO and stent patency. Third, as this was a single‐center study conducted in a specialized tertiary care setting, the generalizability of the findings to broader patient populations or institutions with varying procedural volumes and expertise may be limited. Fourth, the study compared groups with different recruitment periods, which could introduce biases in patient selection and treatment outcomes.

In conclusion, MHSEMS reduces early complications and may be preferable for managing DMBO, especially preoperatively. It may also help prevent severe pancreatitis.

## Conflicts of Interest

The authors declare no conflicts of interest.

## Ethics Statement

The study was conducted in accordance with the principles of the Declaration of Helsinki and approved by the Ethics Committee of Toyonaka Municipal Hospital (2024‐12‐4). The requirement for informed consent was waived because of the retrospective nature of the study.

### Clinical Trial Registration

N/A
